# Prevalence of vitamin D deficiency in adult patients admitted to a psychiatric hospital

**DOI:** 10.1192/bjb.2017.34

**Published:** 2018-06

**Authors:** Dipen Patel, Manjunath Minajagi

**Affiliations:** 1 Leicestershire Partnership NHS Trust, Leicester, UK

## Abstract

**Aims and method:**

Vitamin D deficiency is increasing in the general population, and is linked with physical and mental illness. However, evidence on its prevalence in people with mental illness is limited. This study investigated vitamin D deficiency in 104 adult patients admitted to a psychiatric hospital in the UK.

**Results:**

Forty-nine per cent were vitamin D deficient (serum 25(OH)D <30 nmol/L), and a further 42.3% were vitamin D insufficient (<50 nmol/L). On admission, 8.7% of patients were vitamin D sufficient (>50 nmol/L). There were no statistically significant differences in mean serum vitamin D between different subgroups of mental illness.

**Clinical implications:**

Vitamin D deficiency is highly prevalent among individuals with severe mental illness admitted to hospital. Assessment and treatment of vitamin D deficiency should be considered in in-patients to protect musculoskeletal health. Further epidemiological and intervention studies are needed to investigate the role of vitamin D in the pathophysiology of mental disorders.

**Declaration of interest:**

None.

It has been known for many years that severe vitamin D deficiency increases the risk of metabolic bone disease, particularly rickets and osteomalacia, and that moderate vitamin D deficiency leads to an increased risk of osteoporosis and fractures.^1^ More recently, observational studies have provided growing evidence of an association between vitamin D deficiency and an increased risk for many chronic diseases, including autoimmune diseases, some cancers, cardiovascular disease, infectious disease and type 2 diabetes.^2^ The serum level of 25-hydroxyvitamin D (25(OH)D) is the most accurate reflection of an individual's vitamin D status. The National Osteoporosis Society's guidelines define serum 25(OH)D levels <30 nmol/L as deficient, 30–50 nmol/L as inadequate in some people, and >50 nmol/L as sufficient for almost the whole population.^3^ Vitamin D receptors are found in nearly all tissues of the body, including both neuronal and glial cells in the central nervous system and multiple areas of the human brain, including the prefrontal cortex, hippocampus, cingulate gyrus, thalamus, hypothalamus and substantia nigra, many of which have been implicated in the pathophysiology of mental illnesses such as depression and psychosis.^4^ At a molecular level, vitamin D is known to have numerous roles in nervous system health and disease. Animal models have increased our knowledge and understanding of the mechanisms by which vitamin D deficiency affects brain development and its subsequent influence on adult psychiatric and neurological disease. There is evidence that vitamin D has important roles in neurodevelopment, neuroprotection and neuroplasticity, not only by exerting its biological function directly, but also by influencing the expression of genes at a cellular level.^5^ Evidence on its prevalence in people with mental illness, in the UK, is limited. This study investigated the prevalence of vitamin D deficiency among adult patients being admitted to a psychiatric hospital in the UK, to ascertain how widespread and severe it is among our patients, and to further assess whether there are stronger associations with certain subgroups of illness such as depressive or psychotic disorders.

## Method

This was a cross-sectional study, designed as a pilot, to estimate the prevalence of vitamin D deficiency in patients admitted to a psychiatric hospital, and to ascertain any associations between severity of vitamin D deficiency and severity and subclass of mental illness, in addition to environmental and social demographic factors.

Ethical approval was gained from the National Health Service (NHS) Health Research Authority via the Edgbaston Research Ethics Committee, reference 15/WM/0434. Management permission was sought and gained from the host research and development department at Leicestershire Partnership NHS Trust.

A total of 153 patients were admitted to the general adult wards of the unit, between 17 February 2016 and 23 April 2016, and were eligible for inclusion in the study. One hundred and thirty seven individuals had capacity to consent, of which 104 provided informed written consent. Vitamin D levels were requested alongside standard admission blood tests on serum samples collected by venepuncture. Plasma vitamin D levels of serum samples were analysed in the local pathology laboratory, using Siemens Centaur XP analysers with acridinium ester chemiluminescence technology.

Vitamin D deficiency was defined as a serum 25(OH)D level of below 30 nmol/L, insufficiency was defined as a serum 25(OH)D level of 30 nmol/L or above but less than 50 nmol/L, and vitamin D sufficiency was defined as a serum 25(OH)D level of 50 nmol/L or above.

Primary clinical diagnosis using ICD-10 criteria, ethnicity, gender, age and length of stay were subsequently obtained from participants' electronic in-patient medical records.

Data were initially input and analysed using Microsoft Excel, and further descriptive analysis was conducted on IBM SPSS version 20. The Mann–Whitney U test was used to compare means between different samples.

## Results

A total of 104 participants were included in the study; 51% (*N* = 53) were male and 49% (*N* = 51) were female. As shown in Table 1, the mean age of the cohort was 40.6 years, and the median age was 39.5 with a range from 18 to 79. In terms of ethnicity, 76% (*N* = 79) of participants were White British, 11.5% (*N* = 12) were British Asian, 5.8% (*n* = 6) were Black British, 3.8% (*N* = 4) were of any other White background and 2.9% (*N* = 3) were of other Asian background.

**Table 1:** Demographic and clinical characteristics of the population sample

Demographic and clinical characteristics of population sample (*N* = 104)		
**Age, years**		
Mean	40.6	
Median	39.5	
Range	61	
Minimum	18	
Maximum	79	
**Gender**	**Frequency**	**%**
Female	51	49
Male	53	51
Total	104	100
**Ethnicity**	**Frequency**	**%**
White British	79	76
British Asian	12	11.5
Black British	6	5.8
Any other White Background	4	3.8
Other Asian Background	3	2.9
Total	104	100
**Primary diagnosis**	**Frequency**	**%**
Depressive episode	29	27.9
Bipolar affective disorder	18	17.3
Schizophrenia	18	17.3
Personality disorder	14	13.5
Acute or unspecified psychotic disorder	7	6.7
Psychoactive substance-related disorders	7	6.7
Schizoaffective disorder	5	4.8
Anxiety disorder	3	2.9
Delusional disorder	2	1.9
Eating disorder	1	1
Total	104	100

In the sample studied, mean serum 25(OH)D was 31.9 nmol/L, and median serum 25(OH)D was 32.0 nmol/L, with a range from <15 nmol/L to 81 nmol/L.

There were no statistically significant differences noted in mean serum 25(OH)D associated with gender, age or primary diagnosis. As shown in Table 2, individuals with a primary diagnosis of schizophrenia were noted to have the lowest mean serum 25(OH)D of 29.5 nmol/L, while those with bipolar affective disorder had the highest mean serum 25(OH)D level of 33.8 nmol/L. Mean serum 25(OH)D was higher in participants of White British ethnicity compared with those of other ethnic backgrounds.

**Table 2:** Mean vitamin D in subgroups

	Serum vitamin D, nmol/L	*N*	s.d.
**Whole sample**			
Mean	31.9	104	13.5
Median	32		
Minimum	15		
Maximum	81		
**Gender**			
Female	32.1	51	14.1
Male	31.7	53	13.2
**Primary diagnosis**			
Bipolar affective disorder	33.8	18	17.2
Personality disorder	33.5	14	15.8
Depressive episode	31.4	29	9.4
Schizophrenia	29.5	18	10.3
**Ethnicity**			
White British	33.2	79	13.7
British Asian	28.5	12	13.9
Black British	28.8	6	14
Any other White background	23.5	4	6.2
Other Asian background	28	3	10.4

**Fig. 1:**
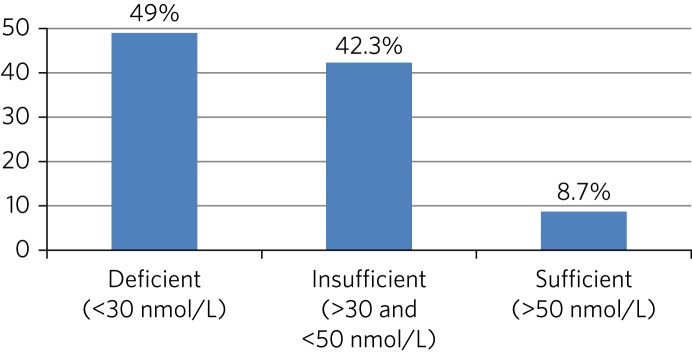
Percentage of participants found to be vitamin D deficient, insufficient and sufficient (*n* = 104).

As shown in Fig. 1, 49% (*N* = 51) of participants were vitamin D deficient (serum 25(OH)D <30 nmol/L), and a further 42% (*N* = 44) were vitamin D insufficient (<50 nmol/L); 8.7% (*N* = 9) of participants were vitamin D sufficient (>50 nmol/L).

Although not statistically significant, mean length of stay in participants with vitamin D deficiency (<30 nmol/L) was 45.4 days, 8 days longer than those without deficiency (*P* = 0.23).

## Discussion

To our knowledge, this is the first study to assess prevalence of vitamin D deficiency in patients admitted to a general adult psychiatry unit in the UK. We found a very high prevalence of vitamin D deficiency in patients with mental illness being admitted to psychiatric hospital, with a total of 49% being deficient and a further 42% being insufficient in vitamin D.

The reported prevalence in our study was greater than that found in psychiatric in-patient samples in Australia (58% less than 50 nmol/L),^6^ New Zealand (74% less than 50 nmol/L)^7^ and North America (52.3% less than 50 nmol/L),^8^ and in an out-patient sample in Northern Europe (56% less than 50 nmol/L).^9^

The prevalence of vitamin D deficiency in our study was higher, and the mean serum vitamin D considerably lower, than in the general population in the UK. Results from the National Diet and Nutrition Survey reported a mean serum 25(OH)D of 34.8 nmol/L and a 39.3% rate of deficiency (<25 nmol/L) in 19–64 year olds between the months of January and March, while individuals 65 years and older had a mean serum 25(OH)D of 40.5 nmol/L and a 29.3% rate of deficiency.^10^

In this study, although the differences were not statistically significant, of the four most common diagnostic groups, we found that participants in our sample with a primary diagnosis of schizophrenia had the lowest mean serum vitamin D of 29.5 nmol/L. This finding has been replicated in other studies. A trend towards lower vitamin D levels in individuals with schizophrenia has been reported in a study of psychiatric out-patients,^11^ while a mini meta-analysis of seven studies found that individuals with schizophrenia had a medium effect size for lower vitamin D compared with healthy controls, and also a trend for lower vitamin D levels in comparison with other psychoses.^12^

The UK has an insufficient intensity of ultraviolet sunlight to enable cutaneous synthesis of vitamin D over the winter months, between October and April, thus resulting in the vitamin D status of the UK population peaking in September but then falling continually until the start of the following summer in May, when levels begin to rise again.^13^ Historically, vitamin D deficiency has been linked to metabolic bone disease, specifically serum concentrations of <20 nmol/L being associated with clinical osteomalacia in adults and rickets in children.^14^

It is unclear why the prevalence of vitamin D deficiency found in this study was higher compared with the general population. In addition to cutaneous synthesis during the summer months, vitamin D can be obtained from the diet, but only a few foods such as fatty fish are good sources of vitamin D.^1^ Although institutionalisation in this case would not be a contributing factor, as the participants were newly admitted, it could be hypothesised that people with mental illness are less likely to be spending time outdoors in sunshine and thus produce less endogenous vitamin D over the summer months.

National Institute for Health and Care Excellence and Department of Health guidance identifies the need for vitamin D supplements to be taken by certain at-risk groups, namely all pregnant and breastfeeding women, particularly teenagers and young women; infants and children under 5 years old and people over 65 years old; people who have low or no exposure to the sun, i.e. housebound individuals; and people who have darker skin, for example, people of African, African–Caribbean and South Asian origin.

In a change to previous advice, Public Health England revised its guidelines in 2016, based on a report by the Scientific Advisory Committee on Nutrition. The report recognised the growing prevalence of vitamin D deficiency and is now recommending a reference nutrient intake for vitamin D of 10 μg/day (400 IU/day) throughout the year, for everyone in the general UK population aged 4 years and above, to ensure that the majority of the UK population has enough vitamin D to protect musculoskeletal health year-round.^15^

Our study has some limitations in terms of its generalisability to the wider population. A total of 104 participants were included, representing a relatively small sample size. Of 153 potential participants, informed consent was obtained from 104 individuals, which could have affected results. As the study was cross-sectional in design, no firm conclusions can be made regarding vitamin D deficiency, mental illness and the direction of causality if present. Our study was also limited to participants being admitted to a single unit. Finally, the study was conducted during the winter months; thus, the reported prevalence of vitamin D deficiency would be affected by sun exposure if the study was conducted towards the end of summer.

Based on this study, in which almost half of the participants admitted to psychiatric hospital were found to be deficient in vitamin D, assessment and treatment of vitamin D deficiency by oral supplementation should be considered to protect musculoskeletal health, alongside other physical health interventions, in patients with mental illness admitted to psychiatric wards.

The NHS Five Year Forward View for mental health has recognised the importance of preventable physical health problems in people with severe mental illness,^16^ and thus musculoskeletal health should be optimised where possible.

At the current time, there is insufficient evidence to draw any firm conclusions regarding an association between vitamin D deficiency and non-musculoskeletal health outcomes, including mental illness. More research in the form of larger epidemiological and intervention studies are needed to investigate the association between vitamin D and mental health outcomes; indeed, randomised controlled trials are planned that will hopefully shed more light on this intriguing area in the future.

## About the authors

**Dipen Patel** is a clinical fellow in Psychiatry and **Manjunath Minajagi** is a Consultant Psychiatrist at Bradgate Mental Health Unit, Leicestershire Partnership NHS Trust, Leicester, UK.
